# International Initiative for Harmonization of Plasma Neurofilament light chain NfL clinical reporting in neurodegenerative diseases

**DOI:** 10.1002/alz.090643

**Published:** 2025-01-09

**Authors:** Constance Delaby, Charlotte Teunissen, Dorte Aalund Olsen, Daniel Alcolea, Alberto Lleo, Alicia Algeciras‐Schimnich, Gustavo A A Santos, Elodie Bouaziz Amar, Xavier Ayrignac, Inês Baldeiras, Edith Bigot‐Corbel, Maria Bjerke, Mercedes Carretero Perez, Tiziana Casoli, Tinatin Chabrashvili, Miles D Chapman, Jessica Cusato, Erdinc Dursun, Anthony Fourier, Daniela Galimberti, Duygu Gezen‐Ak, Brian A. Gordon, Melanie Hart, Marina Herwerth, Flora Kaczorowski, Kensaku Kasuga, Ashvini Keshavan, Michael Khalil, Peter Koertvelyessy, Jens Kuhle, Aurélie Ladang, Piotr Lewczuk, Giancarlo Logroscino, Magda Tsolaki, Guido Maria Giuffrè, Marie‐Céline Blanc, Barbara Mroszko, Giulia Musso, Agnieszka Kulczynska‐Przybik, Léonor Nogueira, Claire Paquet, Piero Parchi, Lucilla Parnetti, Raquel Perez Garay, Koen Poesen, Isabelle Quadrio, Muriel Quillard‐Muraine, Enrique Rodriguez Borja, Susanna Schraen, Daniela Terracciano, Hayrettin Tumani, Socrates and John Tzartos, Nadine Unterwalder, Lisa Vermunt, Cheryl L Wellington, Jose Yriarte, Kaj Blennow, Henrik Zetterberg, Sylvain Lehmann

**Affiliations:** ^1^ Université de Montpellier, Montpellier France; ^2^ VU University Medical Center, Alzheimer center VUmc, Neurocampus, Amsterdam Netherlands; ^3^ University hospital of southern Denmark,, Odense, southern Denmark Denmark; ^4^ Hospital de la Santa Creu i Sant Pau, Barcelona, Barcelona Spain; ^5^ Centro de Investigación Biomédica en Red en Enfermedades Neurodegenerativas, CIBERNED, Barcelona Spain; ^6^ Mayo Clinic, Rochester, MN USA; ^7^ Anhanguera University, São Paulo Brazil; ^8^ Cognitive Neurology Center, Hôpital Lariboisière‐Fernand Widal APHP, Paris France; ^9^ Montpellier university, Montpellier, Occitanie France; ^10^ Center for Innovative Biomedicine and Biotechnology, University of Coimbra, Coimbra Portugal; ^11^ CHU Nantes, Nantes France; ^12^ Vrije Universiteit Brussel (VUB), Brussels Belgium; ^13^ Santa Cruz de Tenerife, Tenerife Spain; ^14^ IRCCS INRCA, Ancona Italy; ^15^ SUNY Upstate Medical University, Syracuse, NY USA; ^16^ Neuroimmunology and CSF laboratory, National Hospital for Neurology and Neurosurgery, Queen Square, London United Kingdom; ^17^ UNITO, Turin Italy; ^18^ Institute of Neurological Sciences, Istanbul University‐Cerrahpasa, Istanbul Turkey; ^19^ HCL, Lyon France; ^20^ University of Milan, Milan Italy; ^21^ Deparment of Neuroscience, Institute of Neurological Sciences, Istanbul University‐Cerrahpasa, Istanbul Turkey; ^22^ Washington University in St. Louis School of Medicine, St. Louis, MO USA; ^23^ Department of Neuroinflammation, UCL Queen Square Institute of Neurology, London United Kingdom; ^24^ USZ, Zurich Switzerland; ^25^ Department of Molecular Genetics, Brain Research Institute, Niigata University, Niigata, Niigata Japan; ^26^ Dementia Research Centre, UCL Queen Square Institute of Neurology, London United Kingdom; ^27^ Medical University of Graz, Graz Austria; ^28^ Charité Universitätsmedizin Berlin, Berlin Germany; ^29^ University Hospital Basel, Basel Switzerland; ^30^ CHUI Liège, Liège Belgium; ^31^ Universitätsklinikum Erlagen, Erlangen Germany; ^32^ University of Bari, Bari Italy; ^33^ AHEPA University Hospital, Thessaloniki Greece; ^34^ University of Padova, Padova Italy; ^35^ APHP Site Cochin, Paris France; ^36^ Department of Neurodegeneration Diagnostics, Medical University of Białystok, Poland, Bialystok Poland; ^37^ Medical University of Bialystok, Bialystok Poland; ^38^ CHU‐PURPAN, Toulouse France; ^39^ Université de Paris Cité, Paris France; ^40^ University of Bologna, Bologna Italy; ^41^ University of Perugia, Perugia Italy; ^42^ Biobizkaia, Bizkaia Spain; ^43^ Alzheimer Research Centre, Leuven Brain Institute, KU Leuven, Leuven Belgium; ^44^ Clinical Biology Center, Rouen University Hospital, Rouen France; ^45^ Valencia, Valencia Spain; ^46^ CHU de Lille, Lille France; ^47^ University Federico II, Naples Italy; ^48^ University of Ulm, Ulm Germany; ^49^ University of Athens, Athens Greece; ^50^ LaborBerlin, Berlin France; ^51^ Alzheimer Center Amsterdam, Neurology, Vrije Universiteit Amsterdam, Amsterdam UMC location VUmc, Amsterdam, North Holland Netherlands; ^52^ University of British Columbia, Vancouver, BC Canada; ^53^ Hospital do Meixoeiro, Meixoeiro Spain; ^54^ University of Gothenburg, Mölndal, Gothenburg Sweden

## Abstract

**Background:**

The quantification of neurofilament light chain (NfL) in blood and cerebrospinal fluid (CSF) has proved useful in many contexts, for the diagnosis and prognosis of various neurological disorders. There is, however, a diversity of practices between centers, essentially linked to the context of use (COU), analytical methods, consideration of comorbidities, determination of cut‐points or use of interpretation scales. Finally, for the same biochemical profile, the interpretation and reporting of results may differ from one center to another, raising the question of test commutability. To date, no consensus has been reached between the different laboratories involved to define the most appropriate conclusions/comments based on COU and cut‐points. This work is an essential step towards consensual harmonization of the clinical use of NfL after CSF and/or blood analysis in various neurological contexts, as advocated by the Alzheimer's Association "Biofluid Based Biomarkers PIA" working group.

**Method:**

This international project involves 58 clinical laboratories in 16 countries, specializing in the biochemical diagnosis of neurological disorders. By means of a questionnaire, we obtained a description of the COU, pre‐analytical and analytical (biological fluid and method used to quantify NfL) protocols of all the centers involved.

**Results:**

Of the centers, 42% quantified NfL in CSF, 29% in serum and 28% in plasma, and 1% in dried blood spot. The COUs were as follows: Frontotemporal dementia (FTD, 17%), Alzheimer's disease (AD, 16%), multiple sclerosis (MS, 16%), amyotrophic lateral sclerosis (ALS, 11%), psychiatric syndrome (PS, 10%), Creutzfeldt‐Jakob disease (CJD, 8%), Parkinson's disease (PD, 8%), peripheral neuropathy (PN, 7%) and traumatic brain injury (TBI, 7%). Most centers define pathological cut‐points based on published literature and take age into account (50%).

**Conclusion:**

Our initial results highlight the state of the art in terms of the clinical use of NfL analysis in CSF and blood in the context of different neurological diseases. We have now defined a coordinator for each COU subgroup and are organizing consensus meetings to harmonize the use and reporting of NfL measurements for the identified clinical applications. The results of these next steps will be presented.

